# Visualizing Biological Copper Storage: The Importance of Thiolate‐Coordinated Tetranuclear Clusters

**DOI:** 10.1002/anie.201703107

**Published:** 2017-06-19

**Authors:** Arnaud Baslé, Semeli Platsaki, Christopher Dennison

**Affiliations:** ^1^ Institute for Cell and Molecular Biosciences, Medical School Newcastle University Newcastle upon Tyne NE2 4HH UK

**Keywords:** bioinorganic chemistry, copper, copper storage, metalloproteins, structural biology

## Abstract

Bacteria possess cytosolic proteins (Csp3s) capable of binding large quantities of copper and preventing toxicity. Crystal structures of a Csp3 plus increasing amounts of Cu^I^ provide atomic‐level information about how a storage protein loads with metal ions. Many more sites are occupied than Cu^I^ equiv added, with binding by twelve central sites dominating. These can form [Cu_4_(S‐Cys)_4_] intermediates leading to [Cu_4_(S‐Cys)_5_]^−^, [Cu_4_(S‐Cys)_6_]^2−^, and [Cu_4_(S‐Cys)_5_(O‐Asn)]^−^ clusters. Construction of the five Cu^I^ sites at the opening of the bundle lags behind the main core, and the two least accessible sites at the opposite end of the bundle are occupied last. Facile Cu^I^ cluster formation, reminiscent of that for inorganic complexes with organothiolate ligands, is largely avoided in biology but is used by proteins that store copper in the cytosol of prokaryotes and eukaryotes, where this reactivity is also key to toxicity.

Important metabolic enzymes in eukaryotes and prokaryotes require copper for their active sites.^1^ To prevent toxicity, eukaryotes store excess cytosolic copper using metallothioneins (MTs).^2^, ^3^, ^4^, ^5^ The ability of bacteria to maintain appreciable amounts of intracellular copper has only recently been discovered.^6^, ^7^ This is achieved by a family of copper storage proteins (the Csps), which are tetramers of four‐helix bundles possessing Cys‐lined cavities binding up to approximately 20 Cu^I^ ions per monomer. The twin‐arginine translocated Csps (*Mt*Csp1 and *Mt*Csp2) from the methanotroph *Methylosinus trichosporium* OB3b are involved in copper storage for the main methane‐oxidizing enzyme.^6^ This organism also possesses a Csp3 (*Mt*Csp3), homologues of which are much more widespread and allow bacteria to accumulate cytosolic copper.^7^ Csp3s may safely store Cu^I^ for currently unknown cytosolic copper enzymes or for export by the copper‐transporting ATPase CopA. *Mt*Csp1 has 13 Cys residues and binds a similar number of Cu^I^ ions,^6^ whereas *Mt*Csp3 has 18 Cys residues and accommodates 19 Cu^I^ ions.^7^ Both bind Cu^I^ via sites with highly novel coordination chemistry. Filling the complete core with metal ions, as in the Csps, has not been observed previously for either naturally occurring or engineered four‐helix bundles.^8^, ^9^, ^10^, ^11^, ^12^ Thiolate‐coordinated Cu^I^ clusters are remarkably rare in biological systems, but the crystallization of *Mt*Csp3 in the presence of increasing amounts of Cu^I^ provides unprecedented insight into how such species form in a protein and are used to drive copper storage.

The crystal structure of *Mt*Csp3 plus ca. 2 molar equiv of Cu^I^ (see Supporting Information^6^, ^7^, ^13^, ^14^, ^15^) has four partially occupied sites (Figure 1 and Table S1 in the Supporting Information). These are Cu11 with an occupancy of 0.25, and Cu12, Cu13, and Cu14, all with occupancies of 0.35, which form a symmetrical tetranuclear cluster (Figure 1 b,c). Cu12 and Cu14 are bound by the Cys residues of CXXXC motifs, that is, from the same α‐helix, whilst Cu11 and Cu13 are ligated by Cys residues on different helices (Cu13 is also weakly coordinated by Asn 58). The thiolates of Cys 97, Cys 101, Cys 114, and Cys 118 bridge between two Cu^I^ ions (μ_2_‐S‐Cys). Additional stabilization is provided by Cu^I^–Cu^I^ interactions (ca. 2.6–2.7 Å) between neighboring metal ions (Figure 1 b). Upon adding ca. 9 equiv, Cu^I^ ions are found at 18 locations in *Mt*Csp3 with a total occupancy of 8.2 (Figure 2 and Table S2 in the Supporting Information). Cu3 to Cu14 have the highest occupancies, with three tetranuclear Cu^I^ clusters present; Cu3–Cu6 and Cu7–Cu10, as well as Cu11–Cu14 (Figure 2 b–d). The addition of ca. 17 equiv of Cu^I^ to *Mt*Csp3 results in binding at 22 sites with a total occupancy of 13.8 (Figure 3 and Table S3 in the Supporting Information). Occupancies increase for all sites in the Cu3–Cu6, Cu7–Cu10, and Cu11–Cu14 clusters, which still constitute the majority of the Cu^I^ core (Figure 3b–d). Alternative forms (Cu5b, Cu7b, Cu9b, and Cu11b) are found at four of the six inter‐helical sites making up Cu3 to Cu14 and the short Cu^I^ to Cu^I^ distances (ca. 1.4 to 1.6 Å) between these and the nearest high occupancy site indicates that both cannot be present within the same molecule (the sum of van der Waals radii is 2.8 Å). These alternate sites contribute to [Cu_4_(μ_2_‐S‐Cys)_4_] clusters (Figures 2 b–d and 3b–d) similar to that observed for Cu11‐14 in the 2 equiv structure (Figure 1 b). However, the major species are [Cu_4_(S‐Cys)_5_]^−^ (Cu3–Cu6), [Cu_4_(S‐Cys)_6_]^2−^ (Cu7–Cu10), and [Cu_4_(S‐Cys)_5_(O‐Asn)]^−^ (Cu11–Cu14), all with three μ_2_‐S(Cys) and either two (Cu3–Cu6 and Cu11–Cu14) or three (Cu7–Cu10) Cys ligands that bind a single Cu^I^ ion at that particular cluster. The major clusters have more ligands and are less symmetrical, highlighted by increased variation in Cu to Cu distances (compare Figure 1 b and 2 b–d). The position of Cu11 in 2 equiv *Mt*Csp3 corresponds to Cu11b in the 8  and 14 equiv structures, which is therefore bound before Cu11a. Cu5b, Cu7b, Cu9b, and Cu11b are all minor species and [Cu_4_(μ_2_‐S‐Cys)_4_] intermediates probably form prior to the final clusters.

**Figure 1:**
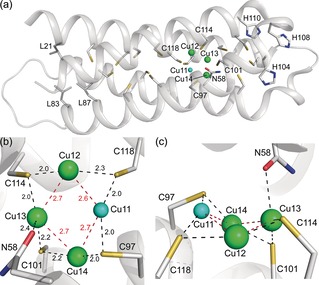
The structure of an *Mt*Csp3 monomer plus approximately 2 equiv of Cu^I^. a) The location of the initial binding sites for Cu^I^ in the four‐helix bundle of *Mt*Csp3 (the N‐terminal helix, α_N_, is omitted). The side chains of all 18 Cys residues, the 3 solvent‐exposed His residues at the mouth and 3 Leu residues at the hydrophobic end of the bundle, and Asn58 are shown as sticks. The size and color (from blue to red for low to high) of the spheres representing the Cu^I^ ions indicate relative occupancy. The structure of the symmetrical Cu11‐Cu14 cluster is shown in detail in (b) and (c). Cu^I^‐ligand bonds and Cu–Cu interactions are shown as black and red dashed lines, respectively, with distances in Å.

**Figure 2:**
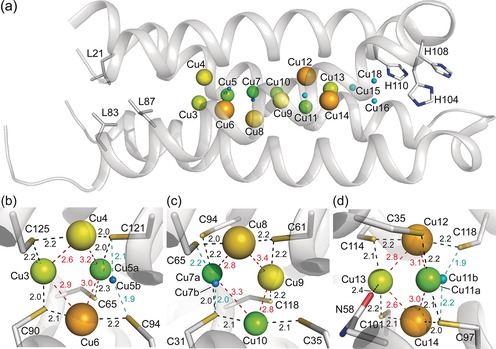
The structure of an *Mt*Csp3 monomer binding 8 equiv of Cu^I^. a) The sites occupied within the core of the bundle (α_N_ omitted). The structures of the Cu3–Cu6, Cu7–Cu10, and Cu11–Cu14 tetranuclear clusters are shown in detail in (b) to (d) respectively. Residues and Cu^I^ ions are represented as described in the legend to Figure 1. The black dashed lines are Cu^I^–ligand bonds (distances in Å) at the major clusters, whilst bonds to the alternate sites, which require coordination by a Cys residue (Cys 94 for Cu5b, Cys 65 for Cu7b, and Cys 118 for Cu11b) that bridges between three Cu^I^ ions (not all at the same cluster), are indicated with cyan dashed lines. The closest Cu^I^ to Cu^I^ distances (Å) for the major sites are shown as red dashed lines.

**Figure 3:**
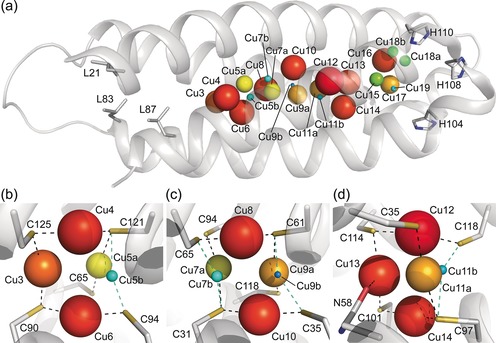
The structure of an *Mt*Csp3 monomer (α_N_ omitted) binding 14 equiv of Cu^I^. The locations of the sites occupied are shown in (a) with detailed structures of the Cu3–Cu6, Cu7–Cu10, and Cu11–Cu14 tetranuclear clusters shown in (b) to (d), respectively. The side chains of key residues, Cu^I^‐ligand bonds and the metal ions are represented as described in the legends to Figures 1 and 2. The Cu^I^‐ligand and Cu^I^ to Cu^I^ distances are similar to those in the 8 equiv structure (see Figure 2 and Tables S2 and S3 in the Supporting Information). An additional alternative site is found at Cu9 (c) whose structure and formation matches that of the other minor forms. Cu1 and Cu2 are not occupied in this structure and although the least accessible are the last to bind Cu^I^.

Cu15 to Cu19 (Figure 4 and Table S3 in the Supporting Information) towards the mouth of the bundle (Figure 1 a) are all partially occupied in 14 equiv *Mt*Csp3 (Cu15, Cu16, and Cu18 are in the 8 equiv structure). Cu18 exists in two equally occupied (0.40) two‐coordinate sites separated by 2.1 Å (not present in the same monomer). Both are ligated by His 110, with either Cys111 (Cu18a) or Cys 38 (Cu18b) as the second ligand. Cu18a corresponds to the site in the fully Cu^I^‐loaded (19 equiv) structure (Figure 4 c and Table S4 in the Supporting Information), whilst Cu18b is occupied in the 8 equiv structure (Figure 4 a), and Cu^I^ ions bind at Cu18b before Cu18a. Cu19 is relocated by more than 1 Å in the 14 equiv compared to 19 equiv structure (Figure 4 b,c), being more distant from His104 (the imidazole of His104 rotates by ca. 180° in the 19 equiv structure allowing its N^δ1^ atom to coordinate Cu19). Cu17 is 1.8 Å from Cu19 in the 14 equiv structure and both cannot be occupied in the same molecule. Cu15 is two‐coordinate in the 8 equiv and 14 equiv structures, whilst in 19 equiv *Mt*Csp3 it is the only site ligated by three Cys ligands,^7^ primarily due to altered conformations of Cys 101 and Cys 111. Five Cys and two His residues rearrange to accommodate the Cu15‐Cu19 cluster at the mouth of the four‐helix bundle as *Mt*Csp3 fills with Cu^I^.

**Figure 4:**
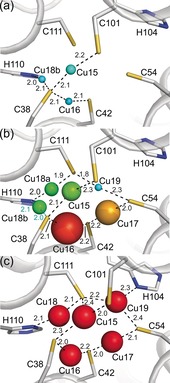
Filling of the Cu15 to Cu19 sites at the mouth of the *Mt*Csp3 four‐helix bundle. The changes in this region in the a) 8 equiv, b) 14 equiv, and c) 19 equiv structures are shown. The side chains of key residues, Cu^I^‐ligand bonds (distances in Å), and the Cu^I^ ions in (a) and (b) are represented as described in the legends to Figures 1 and 2. Cu16 has the highest occupancy (0.90) in the 14 equiv structure (b), probably as it is the only site in this region bound by a CXXXC motif, whereas all of the Cu^I^ sites in (c) are fully occupied, but are represented using a smaller sphere radius for clarity.

More sites are occupied than the number of Cu^I^ equiv added in all of the *Mt*Csp3 structures. Furthermore, no site is fully occupied until the 14 equiv structure (Cu12), and there is a general tendency that Cu^I^ favors binding at CXXXC motifs. Cu^I^ ions populate numerous sites as the bundle fills, with a clear preference for those towards the center, and particularly in the Cu3–Cu6, Cu7–Cu10, and Cu11–Cu14 tetranuclear clusters (Figure S1 in the Supporting Information). This behavior is consistent with in vitro Cu^I^‐binding properties,^7^ and a more ordered uptake mechanism for *Mt*Csp3 compared to *Mt*Csp1 (for further discussion see the Supporting Information). The total occupancies of the Cu3–Cu6, Cu7–Cu10, and Cu11–Cu14 clusters range from 2.4 to 2.7 in the 8 equiv structure and increase to 3.5 to 3.7 in 14 equiv *Mt*Csp3 (Figures 2, 3, and Figure S2 and Tables S2, S3 in the Supporting Information). There is no preference for Cu^I^ binding at a particular tetranuclear cluster apart from in the structure plus ca. 2 equiv, in which only Cu11–Cu14 is occupied. This must be due to the fact that as Cu^I^ ions diffuse past Cu15 to Cu19 at the mouth of the bundle, Cu11–Cu14 are the first sites they encounter at which they can form a tetranuclear cluster. The three tetranuclear clusters probably have similar Cu^I^‐binding affinities, and occupancy of the Cu3–Cu14 core appears thermodynamically favored over other sites.

Copper clusters in proteins are extremely rare, with the Cu_Z_ site of nitrous oxide reductase providing the only example of a tetranuclear site in an enzyme.^16^ However, this site lacks any Cys ligands, and the Cu_A_ center, also found in nitrous oxide reductase as well as cytochrome oxidases, is the highest nuclearity copper site involving Cys ligands in an enzyme (Cu_2_(S‐Cys)_2_(*N*‐His)_2_).^16^, ^17^ The scarcity of Cu^I^ clusters bound by organothiolates in biological systems, such as those that drive *Mt*Csp3 Cu^I^ core formation, is surprising given their rich coordination chemistry^18^, ^19^ and the thiophilic nature of Cu^I^. Proteins involved in copper homeostasis, particularly in *Saccharomyces cerevisiae*, have been found to bind tetranuclear Cu^I^ clusters in vitro using Cys residues, but no crystal structures are available.^20^, ^21^, ^22^, ^23^, ^24^ Extended X‐ray absorption fine structure data for these are similar to that of [Cu_4_(SPh)_6_]^2−^.^20^, ^21^, ^23^, ^25^ In this complex the Cu^I^ ions are three‐coordinate and all thiolates μ_2_‐S.^18^ The crystal structure of a side‐to‐side dimer of a cyanobacterial Atx1^13^ binds a symmetrical [Cu_4_(μ_2_‐S‐Cys)_4_Cl_2_]^2−^ cluster comparable to the [Cu_4_(μ_2_‐S‐Cys)_4_] intermediates we observe for *Mt*Csp3, but a functional role for this Atx1 dimer remains to be established. Thiolate‐coordinated Cu^I^‐cluster formation is physiologically important in the MTs. These, almost exclusively eukaryotic, Cys‐rich unstructured apo‐polypeptides store cytosolic Cu^I^ by folding around clusters.^2^, ^3^, ^4^, ^5^ The crystal structure of a truncated *S. cerevisiae* MT (Cup1) has eight Cu^I^ ions bound by ten Cys residues, with the majority of sites coordinatively saturated (three‐coordinate).^4^ These are present as two Cu^I^_4_ flattened tetrahedra, each with structures similar to [Cu_4_(SPh)_6_]^2−^.^18^ The presence of linked tetranuclear clusters in the MT structure is comparable to what we have observed for the Cu3 to Cu14 core of *Mt*Csp3, but most sites in the Csps are two‐coordinate. This is partly due to a low Cys:Cu^I^ ratio in the Csps (always about 1) and their four‐helix bundle fold that prevents significant re‐positioning of the Cys residues. Maintaining two‐coordinate sites in Csps may be important either to facilitate Cu^I^ release or for the safe use of Cys residues for binding Cu^I^ clusters.

The avoidance of copper clusters in enzymes is probably due to the enhanced risk of toxicity. This could be exacerbated by the presence of Cys ligands, due to the potential for Cu^II^‐catalyzed disulfide bond formation (a dedicated protein is required to keep the Cys residues reduced prior to copper insertion at the Cu_A_ site^26^). Biologically important Cys‐bound Cu^I^ clusters are found in cytosolic copper storage proteins (Csp3s and MTs) in which disulfides do not occur. In both cases, the reducing nature of the cytosol helps maintain thiols (exported Csps fold in the cytosol^6^). The rigidity of the four‐helix bundle fold also prevents this reactivity by geometrically constraining Cys residues in the Csps (the Cys residues of apo‐Csps do not readily form disulfide bonds in air^6^, ^7^), as well as providing additional protection of bound Cu^I^ ions. This is not the case for the unstructured and flexible MTs.

In conclusion, we provide a visual description of Cu^I^ loading in a storage protein, highlighting factors important for the formation of the Cu^I^ core, as well as the fluxionality and flexibility of Cu^I^ binding. We identify [Cu_4_(μ_2_‐S‐Cys)_4_] as an intermediate for the three clusters that are made up of the first sites (Cu3 to Cu14) to be occupied in *Mt*Csp3. The ability to store and protect Cu^I^ ions in the cytosol is driven by the formation of thiolate‐coordinated tetranuclear clusters in both prokaryotes and eukaryotes, but is achieved with dramatically different protein structures. Paradoxically, the same facile cluster chemistry of Cu^I^ is also responsible for toxicity by the displacement of iron at Cys‐bound 4Fe–4S clusters.^27^, ^28^, ^29^, ^30^ MTs have been suggested to contribute to a “chelation” rather than “compartmentalization” mechanism to combat copper toxicity in eukaryotes.^27^ Csp3‐possessing bacteria have a cytosolic detoxification system for copper,^7^ which also relies on chelation. Thus copper handling by such organisms is more complex than originally thought and requires further investigation.

## Conflict of interest

The authors declare no conflict of interest.

## Supporting information

As a service to our authors and readers, this journal provides supporting information supplied by the authors. Such materials are peer reviewed and may be re‐organized for online delivery, but are not copy‐edited or typeset. Technical support issues arising from supporting information (other than missing files) should be addressed to the authors.

Supplementary
