# Supplementary material for: Visualizing Biological Copper Storage: The Importance of Thiolate‐Coordinated Tetranuclear Clusters
Source: Angew Chem Int Ed Engl. 2017 Jun 19;56(30):8697–700. doi: 10.1002/anie.201703107 (PMC5519932; doi:10.1002/anie.201703107)
Supplement: Supplementary file 1 — Supplementary [file ANIE-56-8697-s001.pdf]

Angewandte Chemie
Eine Zeitschrift der Gesellschaft Deutscher Chemiker

Supporting Information

Visualizing Biological Copper Storage: The Importance of Thiolate-Coordinated Tetranuclear Clusters

*Arnaud Baslé, Semeli Platsaki, and Christopher Dennison**

anie_201703107_sm_miscellaneous_information.pdf

Table of Contents

Materials and Methods 2

Additional Discussion: Importance of the Structures for the *in vitro* Cu(I) Binding Properties of Csps 3

Figure S1. Core formation in a copper storage protein 4

Figure S2. Tetranuclear cluster formation in *Mt*Csp3 5

Figure S3. A comparison of the Cu(I) cores of *Mt*Csp3 and *Mt*Csp1 6

Table S1: The Cu(I) sites in the structure of *Mt*Csp3 binding 1-2 eq. of Cu(I) 7

Table S2: The Cu(I) sites in the structure of *Mt*Csp3 binding 8 eq. of Cu(I) 8

Table S3: The Cu(I) sites in the structure of *Mt*Csp3 binding 14 eq. of Cu(I) 9

Table S4: The Cu(I) sites in the structure of *Mt*Csp3 binding 19 eq. of Cu(I) 10

Table S5: Data collection and refinement statistics 11

References 12

Materials and Methods

Protein Purification, Cu(I) Loading and Crystallization

Apo-*Mt*Csp3 was purified and quantified, and Cu(I)-*Mt*Csp3 samples prepared, as described previously.[1,2] Cu(I) stock solutions (typically ~100 mM [Cu(CH_3CN)$_4$]PF_6 in 100% acetonitrile) were diluted to ~1-10 mM in 20 mM Hepes pH 7.5 plus 200 mM NaCl in an anaerobic chamber (Belle Technology, [O_2] <<2 ppm).[1-5] The Cu(I) concentration of the diluted Cu(I) solution in buffer was quantified anaerobically with the chromophoric high affinity Cu(I) ligand bathocuproine disulfonate (BCS) by monitoring formation of $[Cu(BCS)_2]^{3-}$ at 483 nm (ε = 12500 M^{-1} cm^{-1}).[1-5] Cu(I) additions were based on apo-*Mt*Csp3 concentrations determined using the 5,5'-dithiobis(2-nitrobenzoic acid) (Ellman's reagent) assay. Apo-*Mt*Csp3 (70 μM) was mixed with approximately 2 or 9 molar eq. of Cu(I), whilst a more dilute apo-protein (53 μM) sample was incubated with approximately 17 eq. of Cu(I), all in 20 mM Hepes pH 7.5 plus 200 mM NaCl. Samples were concentrated using ultrafiltration, and all procedures were performed in the anaerobic chamber. Cu(I)-*Mt*Csp3 (~9.8, 12 and 13.5 mg/mL by Bradford assay, to which ~2, 9 and 17 eq. of Cu(I) respectively had been added) were crystallized using the sitting drop method of vapor diffusion. Cu(I)-protein samples were removed from the anaerobic chamber to use a crystallization robot, and trays were transferred back to the chamber as quickly as possible and sealed (Cu(I)-*Mt*Csp3 exposed to oxygen for approximately 20 mins). Diffraction-quality crystals of protein to which 2 and 9 eq. of Cu(I) were added formed from protein (600 nl) mixed with 300 nl of 200 mM $MgCl_2$, 100 mM Hepes pH 7.5, 30% PEG 400 (80 μL well volume) and were frozen directly. Diffraction-quality crystals of *Mt*Csp3 to which 17 eq. of Cu(I) had been added were obtained using the same condition (100 nl) mixed with 200 nl of protein (80 μl well volume) and were frozen directly.

Data Collection, Structure Solution and Refinement

All crystallographic data were collected at Diamond Light Source Ltd, UK, beamline I02. Data were integrated either with iMosflm or XDS,[6,7] scaled with Aimless[8] and space group determination was confirmed with Pointless.[9] Structures were solved by molecular replacement using Molrep implemented via the CCP4 suite[10] with apo-*Mt*Csp3 (5ARM)[2] as the search model. Cycles of model building in Coot[11] and refinement in REFMAC5[12] were performed. Occupancies of copper sites were adjusted manually in 5% increments based on observed peaks in difference maps. The agreement between total copper occupancies in structures and the number of Cu(I) eq. added is within the errors of determinations using these two approaches. Five percent of observations were used to monitor refinement. All models were validated using MolProbity[13] and data collection statistics and refinement details are reported in Table S5.

Additional Discussion

Importance of the Structures for the *in vitro* Cu(I) Binding Properties of Csps

Upon the addition of Cu(I), *Mt*Csp3 gives rise to fluorescence at around 600 nm,[2] which reaches a maximum at ~9-11 eq.. This emission must be due to the three buried[14-17] thiolate-coordinated tetranuclear clusters (Figure 2). Further Cu(I) binding causes emission to decrease, probably due to the population of the solvent accessible sites[14-17] near the mouth of the bundle (Figure 3). Tetranuclear Cu(I) clusters like those seen in *Mt*Csp3 are not present in *Mt*Csp1,[1] due to the absence (Figure S3) of at least one of the Cys ligands required (*Mt*Csp1 has 13 Cys residues compared to 18 in *Mt*Csp3 and only binds 13 Cu(I) ions). Thus *Mt*Csp1 exhibits little emission at 600 nm upon Cu(I) binding.[1] The relatively independent formation in *Mt*Csp3 of the three tetranuclear Cu(I) clusters, the Cu15-Cu19 arrangement at the mouth of the bundle and the final two Cu(I) sites (Cu1 and Cu2), results in overall non-cooperative Cu(I) binding (cooperativity within the Cu3-Cu14 core is possible).[2] A more disordered core-formation mechanism is likely to be operative in *Mt*Csp1, with Cu(I) ions accessing a greater proportion of sites within the core (initial NMR studies indicate this to be the case), giving rise to overall positive cooperativity.[1] These different core-formation, and presumably also release, mechanisms must contribute to the very different Cu(I) removal kinetics for *Mt*Csp3 and *Mt*Csp1. For example, a large excess of the high affinity ligand BCS can completely strip *Mt*Csp1 of Cu(I) in ~30 min,[1] whereas this process is much slower for *Mt*Csp3 under identical conditions, occurring over days.[2]

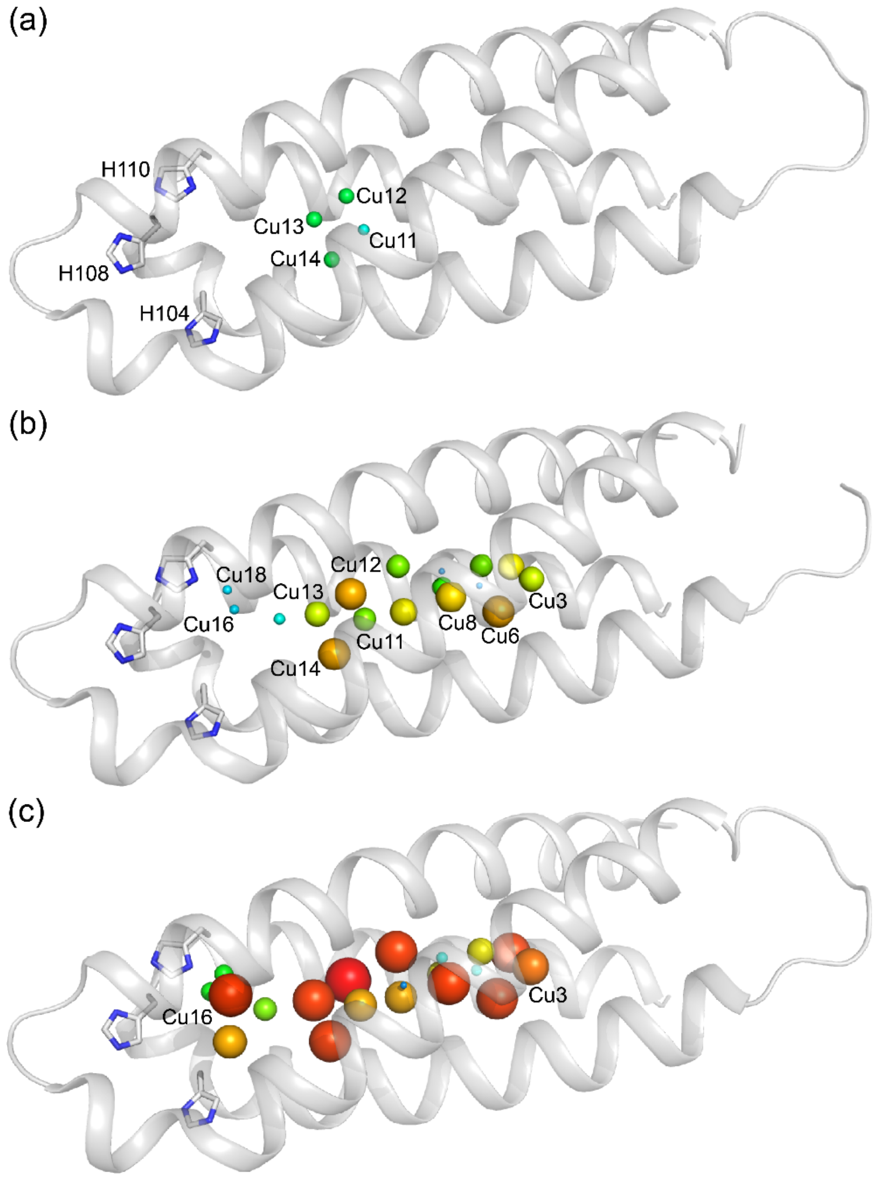

Figure S1. Core formation in a copper storage protein. The structures of an *Mt*Csp3 monomer with the N-terminal helix (α_N) omitted binding 1-2 eq. (a), 8 eq. (b) and 14 eq. (c) of Cu(I). The three His residues at the mouth of the four-helix bundle are shown as sticks. The size and colour (from blue to red for low to high) of the spheres representing the Cu(I) ions indicate relative occupancy.

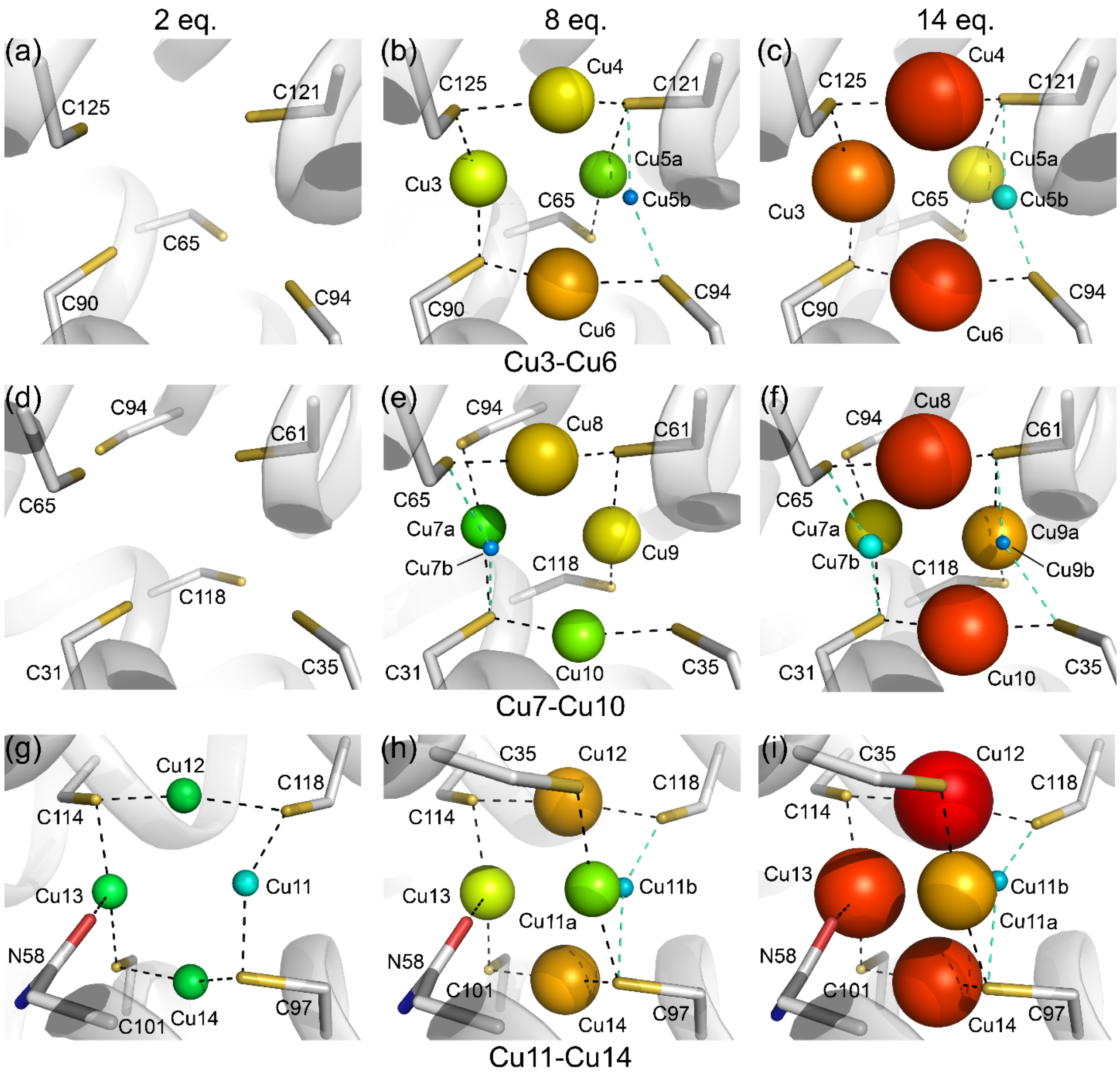

Figure S2. Tetranuclear cluster formation in *Mt*Csp3. The structures of the Cu3-Cu6 (a-c), Cu7-Cu10 (d-f) and Cu11-Cu14 (g-i) clusters in *Mt*Csp3 binding 1-2 eq. (a, d, g), 8 eq. (b, e, h) and 14 eq. (c, f, i) of Cu(I). The coordinating residues (Cys residues as well as Asn58 at Cu11-Cu14) are shown as sticks. The size and colour (from blue to red for low to high) of the spheres representing the Cu(I) ions indicate relative occupancy. The Cu–ligand bonds at the major clusters are shown as black dashed lines, whilst bonds to the alternate Cu(I) sites are shown as dashed cyan lines. Detailed structural information about the sites is provided in Tables S1-S3.

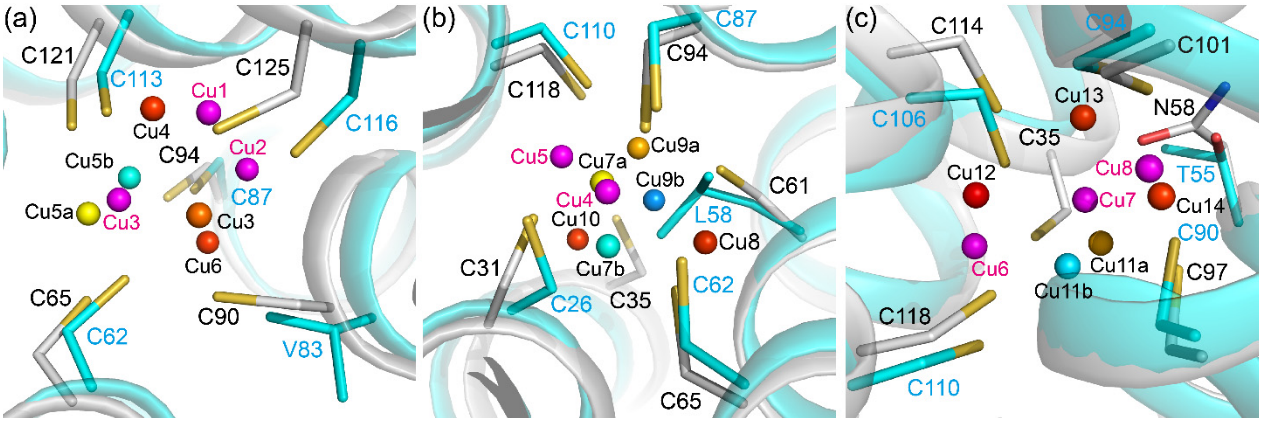

Figure S3. A comparison of the Cu(I) cores of *Mt*Csp3 and *Mt*Csp1. The structures of the Cu3-Cu6 (a), Cu7-Cu10 (b) and Cu11-Cu14 (c) clusters of *Mt*Csp3 binding 14 eq. of Cu(I) (protein shown in light grey) overlaid with the corresponding sites of fully Cu(I)-loaded *Mt*Csp1[1] (protein shown in cyan). The colors of the spheres representing the Cu(I) ions in *Mt*Csp3 (from blue to red for high to low) indicate relative occupancy, whilst all Cu(I) ions in *Mt*Csp1 are assumed to have an occupancy of 1 and are shown as magenta spheres (for clarity all sphere sizes are the same regardless of occupancy). Cu(I) ions and ligands are labelled black for *Mt*Csp3 and cyan and magenta respectively in *Mt*Csp1. The presence of non-coordinating residues in *Mt*Csp1 in the locations occupied by Cys ligands in *Mt*Csp3 prevents the formation of tetranuclear clusters. These are Val83 (*Mt*Csp1) in place of Cys90 at Cu3-Cu6 (a), Gly30 and Leu58 (*Mt*Csp1) in place of Cys35 and Cys61 at Cu7-Cu10 (b) and Gly30 (*Mt*Csp1) in place of Cys35 at Cu11-Cu14, at which Thr55 (*Mt*Csp1) is also found in place of the coordinating Asn58 (c).

Table S1: The Cu(I) sites in the structure of *Mt*Csp3 binding 1-2 eq. of Cu(I).[a]

Cu site (occupancy)	Ligands (Cu-ligand distance (Å)	CXXXC/inter-helix
11 (0.25)	S^{γ}(C97, α3) = 2.0, S^{γ}(C118, α4) = 2.0	Inter-helix
12 (0.35)	S^{γ}(C114, α4) = 2.0, S^{γ}(C118, α4) = 2.3, O(C114, α4) = 2.5	CXXXC
13 (0.35)	S^{γ}(C101, α3) = 2.2, S^{γ}(C114, α4) = 2.0, $O^{\delta1}$(N58, α2) = 2.4	Inter-helix
14 (0.35)	S^{γ}(C97, α3) = 2.0, S^{γ}(C101, α3) = 2.2, O(C97, α3) = 2.4	CXXXC

[a] Occupancies, coordinating residues, Cu–ligand bond distances and whether the Cys ligands are either part of a CXXXC motif or arise from different α-helices (helix number shown after the coordinating residue) are indicated. Data taken from PDB file 5NQM.

Table S2: The Cu(I) sites in the structure of *Mt*Csp3 binding 8 eq. of Cu(I).[a]

Cu site (occupancy)	Ligands (Cu-ligand distance (Å)	CXXXC/inter-helix
3 (0.55)	S^{γ}(C90, α3) = 2.0, S^{γ}(C125, α4) = 2.2	Inter-helix
4 (0.60)	S^{γ}(C121, α4) = 2.0, S^{γ}(C125, α4) = 2.2, O(C121, α4) = 2.3	CXXXC
5a (0.50)	S^{γ}(C65, α2) = 2.3, S^{γ}(C121, α4) = 2.0	Inter-helix
5b (0.15)	S^{γ}(C94, α3) = 1.9, S^{γ}(C121, α4) = 2.1	Inter-helix
6 (0.70)	S^{γ}(C90, α3) = 2.1, S^{γ}(C94, α3) = 2.2, O(C90, α3) = 2.4	CXXXC
7a (0.45)	S^{γ}(C31, α1) = 2.0, S^{γ}(C94, α3) = 2.2	Inter-helix
7b (0.15)	S^{γ}(C31, α1) = 2.0, S^{γ}(C65, α2) = 2.2	Inter-helix
8 (0.65)	S^{γ}(C61, α2) = 2.2, S^{γ}(C65, α2) = 2.0, O(C61, α2) = 2.6	CXXXC
9 (0.60)	S^{γ}(C61, α2) = 2.2, S^{γ}(C118, α4) = 2.2	Inter-helix
10 (0.50)	S^{γ}(C31, α1) = 2.1, S^{γ}(C35, α1) = 2.1, O(C31, α1) = 2.3	CXXXC
11a (0.50)	S^{γ}(C35, α1) = 2.2, S^{γ}(C97, α3) = 2.1, $O^{\delta1}$(N58, α2) = 2.6	Inter-helix
11b (0.20)	S^{γ}(C97, α3) = 2.2, S^{γ}(C118, α4) = 1.9	Inter-helix
12 (0.70)	S^{γ}(C114, α4) = 2.2, S^{γ}(C118, α4) = 2.2, O(C114, α4) = 2.4	CXXXC
13 (0.55)	S^{γ}(C101, α3) = 2.2, S^{γ}(C114, α4) = 2.1, $O^{\delta1}$(N58, α2) = 2.4	Inter-helix
14 (0.70)	S^{γ}(C97, α3) = 2.0, S^{γ}(C101, α3) = 2.1, O(C97, α3) = 2.4	CXXXC
15 (0.25)	S^{γ}(C38, α1) = 2.1, S^{γ}(C101, α3) = 2.2	Inter-helix
16 (0.20)	S^{γ}(C38, α1) = 2.1, S^{γ}(C42, α1) = 2.1, O(C38, α1) = 2.4	CXXXC
18 (0.20)	S^{γ}(C38, α1) = 2.1, $N^{\delta1}$(H110, α4) = 2.0	Inter-helix

[a] Occupancies, coordinating residues, Cu–ligand bond distances and whether the Cys ligands are either part of a CXXXC motif or arise from different α-helices (helix number shown after the coordinating residue) are indicated. Data taken from PDB file 5NQN.

Table S3: The Cu(I) sites in the structure of *Mt*Csp3 binding 14 eq. of Cu(I).[a]

Cu site (occupancy)	Ligands (Cu-ligand distance (Å)	CXXXC/inter-helix
3 (0.80)	S^{γ}(C90, α3) = 2.1, S^{γ}(C125, α4) = 2.2	Inter-helix
4 (0.90)	S^{γ}(C121, α4) = 2.1, S^{γ}(C125, α4) = 2.2, O(C121, α4) = 2.2	CXXXC
5a (0.60)	S^{γ}(C65, α2) = 2.2, S^{γ}(C121, α4) = 2.1	Inter-helix
5b (0.25)	S^{γ}(C94, α3) = 1.9, S^{γ}(C121, α4) = 2.2	Inter-helix
6 (0.90)	S^{γ}(C90, α3) = 2.2, S^{γ}(C94, α3) = 2.1, O(C90, α3) = 2.3	CXXXC
7a (0.60)	S^{γ}(C31, α1) = 2.2, S^{γ}(C94, α3) = 2.1	Inter-helix
7b (0.25)	S^{γ}(C31, α1) = 2.1, S^{γ}(C65, α2) = 2.3	Inter-helix
8 (0.90)	S^{γ}(C61, α2) = 2.2, S^{γ}(C65, α2) = 2.1, O(C61, α2) = 2.4	CXXXC
9a (0.70)	S^{γ}(C61, α2) = 2.2, S^{γ}(C118, α4) = 2.2	Inter-helix
9b (0.15)	S^{γ}(C35, α1) = 2.2, S^{γ}(C61, α2) = 2.0	Inter-helix
10 (0.90)	S^{γ}(C31, α1) = 2.1, S^{γ}(C35, α1) = 2.1, O(C31, α1) = 2.3	CXXXC
11a (0.70)	S^{γ}(C35, α1) = 2.2, S^{γ}(C97, α3) = 2.1, $O^{\delta1}$(N58, α2) = 2.6	Inter-helix
11b (0.20)	S^{γ}(C97, α3) = 2.3, S^{γ}(C118, α4) = 1.8	Inter-helix
12 (1.00)	S^{γ}(C114, α4) = 2.2, S^{γ}(C118, α4) = 2.2, O(C114, α4) = 2.3	CXXXC
13 (0.90)	S^{γ}(C101, α3) = 2.2, S^{γ}(C114, α4) = 2.2, $O^{\delta1}$(N58, α2) = 2.3	Inter-helix
14 (0.90)	S^{γ}(C97, α3) = 2.1, S^{γ}(C101, α3) = 2.2, O(C97, α3) = 2.3	CXXXC
15 (0.50)	S^{γ}(C38, α1) = 2.2, S^{γ}(C101, α3) = 2.3	Inter-helix
16 (0.90)	S^{γ}(C38, α1) = 2.1, S^{γ}(C42, α1) = 2.2, O(C38, α1) = 2.3	CXXXC
17 (0.70)	S^{γ}(C42, α1) = 2.2, S^{γ}(C54, α2) = 2.1	Inter-helix
18a (0.40)	$N^{\delta1}$(H110, α4) = 2.0, S^{γ}(C111, α4) = 1.9	*b*
18b (0.40)	S^{γ}(C38, α1) = 2.0, $N^{\delta1}$(H110, α4) = 2.1	Inter-helix
19 (0.20)	S^{γ}(C54, α2) = 2.2, S^{γ}(C111, α4) = 1.9	Inter-helix

[a] Occupancies, coordinating residues, Cu–ligand bond distances and whether the Cys ligands are either part of a CXXXC motif or arise from different α-helices (helix number shown after the coordinating residue) are indicated. *b* The His and Cys ligands at Cu18a are adjacent and are therefore on the same helix. Data taken from PDB file 5NQO.

Table S4: The Cu(I) sites in the structure of *Mt*Csp3 binding 19 eq. of Cu(I).[a]

Cu site	Ligands (Cu-ligand distance (Å)	CXXXC/inter-helix
1	S^{γ}(C24, α1) = 2.2, S^{γ}(C28, α1) = 2.2, O(C24, α1) = 2.0	CXXXC
2	S^{γ}(C24, α1) = 2.1, S^{γ}(C125, α4) = 2.2	Inter-helix
3	S^{γ}(C28, α1) = 2.0, S^{γ}(C90, α3) = 2.2	Inter-helix
4	S^{γ}(C121, α4) = 2.1, S^{γ}(C125, α4) = 2.1, O(C121, α4) = 2.4	CXXXC
5a	S^{γ}(C65, α2) = 2.2, S^{γ}(C121, α4) = 2.2	Inter-helix
6	S^{γ}(C90, α3) = 2.2, S^{γ}(C94, α3) = 2.2, O(C90, α3) = 2.2	CXXXC
7a	S^{γ}(C31, α1) = 2.0, S^{γ}(C94, α3) = 2.1	Inter-helix
8	S^{γ}(C61, α2) = 2.0, S^{γ}(C65, α2) = 2.2, O(C61, α2) = 2.4	CXXXC
9a	S^{γ}(C61, α2) = 2.2, S^{γ}(C118, α4) = 2.4	Inter-helix
10	S^{γ}(C31, α1) = 2.2, S^{γ}(C35, α1) = 2.2, O(C31, α1) = 2.1	CXXXC
11a	S^{γ}(C35, α1) = 2.1, S^{γ}(C97, α3) = 2.1, $O^{\delta1}$(N58, α2) = 2.7	Inter-helix
12	S^{γ}(C114, α4) = 1.9, S^{γ}(C118, α4) = 2.0, O(C114, α4) = 2.4	CXXXC
13	S^{γ}(C101, α3) = 2.2, S^{γ}(C114, α4) = 2.2, $O^{\delta1}$(N58, α2) = 2.4	Inter-helix
14	S^{γ}(C97, α3) = 2.3, S^{γ}(C101, α3) = 2.4, O(C97, α3) = 2.2	CXXXC
15	S^{γ}(C38, α1) = 2.3, S^{γ}(C101, α3) = 2.0, S^{γ}(C111, α3) = 2.4	Inter-helix
16	S^{γ}(C38, α1) = 2.0, S^{γ}(C42, α1) = 2.3, O(C38, α1) = 2.2	CXXXC
17	S^{γ}(C42, α1) = 2.0, S^{γ}(C54, α2) = 2.1	Inter-helix
18a	$N^{\delta1}$(H110, α4) = 2.1, S^{γ}(C111, α4) = 2.1	*b*
19	S^{γ}(C54, α2) = 2.4, $N^{\delta1}$(H104) = 2.3, S^{γ}(C111, α4) = 2.2	Inter-helix

[a] Coordinating residues, Cu–ligand bond distances and whether the Cys ligands are either part of a CXXXC motif or arise from different α-helices (helix number shown after the coordinating residue) are all indicated. Data taken from PDB file 5ARN and all sites are assumed to be fully occupied. *b* The His and Cys ligands at Cu18a are adjacent and are therefore on the same helix.

Table S5: Data collection and refinement statistics.

	1-2 eq. Cu(I)-*Mt*Csp3<br>5NQM	8 eq. Cu(I)-*Mt*Csp3<br>5NQN	14 eq. Cu(I)-*Mt*Csp3<br>5NQO
**Data collection**[a]			
Beamline	I03	I03	I02
Date	04/10/15	04/10/15	26/07/13
Wavelength (Å)	1.3746	1.3669	0.9796
Resolution (Å)	45.62 – 1.59 (1.62 – 1.59)	45.60 – 1.62 (1.65 – 1.62)	45.62 - 1.15 (1.17 - 1.15)
Space group		C222	
Unit-cell parameters			
a, b, c (Å)	44.7, 106.7, 45.6	44.9, 106.1, 45.6	44.9, 105.7, 45.6
$\alpha = \beta = \gamma$ (°)		90	
Unit-cell volume (Å^3)	217299	217300	216519
Solvent content (%)	34	34	40
No. of measured reflections	53992 (2319)	59025 (2743)	139378 (6853)
No. of independent reflections	14862 (702)	14074 (669)	38830 (1908)
Completeness (%)	99.3 (99.3)	98.7 (92.5)	99.6 (99.7)
Redundancy	3.6 (3.3)	4.2 (4.1)	3.6 (3.6)
R_{merge} (%)	8.3 (38.2)	6.6 (41.4)	5.2 (51.6)
$\langle I \rangle / \langle \sigma(I) \rangle$	7.8 (1.8)	11.6 (3.0)	11.5 (2.3)
**Refinement statistics**[a]			
R_{work} (%)	15.00	14.33	12.15
R_{free}[b] (%)	20.65	21.08	14.54
No. of atoms			
Protein	938	936	995
Solvent	38	70	77
Cu	4	18	23
Average B factors (Å^2)			
Protein	20.5	24.4	14.9
Solvent	24.1	36.8	23.0
Cu	17.4	22.6	10.9
R.m.s. deviation from ideal values			
Bond angle (°)	1.72	1.51	1.62
Bond length (Å)	0.018	0.014	0.014
Ramachandran plot,[c] residues in most favoured regions (%)	100	100	100

[a] Values in parenthesis are for the highest resolution shell. [b] 5% of the randomly selected reflections excluded from refinement. [c] Calculated using MolProbity.[13]

References

[1] N. Vita, S. Platsaki, A. Baslé, S. J. Allen, N. G. Paterson, A. T. Crombie, J. C. Murrell, K. J. Waldron, C. Dennison, *Nature* **2015**, *525*, 140.

[2] N. Vita, G. Landolfi, A. Baslé, S. Platsaki, J. Lee, K. J. Waldron, C. Dennison, *Sci. Rep.* **2016**, *6*, 39065.

[3] A. Badarau, S. J. Firbank, A. A. McCarthy, M. J. Banfield, C. Dennison, *Biochemistry* **2010**, *49*, 7798–7810.

[4] A. Badarau, C. Dennison, *J. Am. Chem. Soc.* **2011**, *133*, 2983–2988.

[5] S. Allen, A. Badarau, C. Dennison, *Biochemistry* **2012**, *51*, 1439–1448.

[6] G. W. Leslie, H. R. Powell, Evolving methods for macromolecular crystallography. **2007**, *245*, 41-51 ISBN 978-1-4020-6314-5.

[7] W. Kabsch, *Acta Cryst*. **2010**, *D66*, 125.

[8] P. R. Evans, G. N. Murshudov, *Acta Cryst.* **2013**, *D69*, 1204.

[9] P. R. Evans, *Acta Cryst*. **2006**, *D62*, 72.

[10] M. D. Winn, C. C. Ballard, K. D. Cowtan, E. J. Dodson, P. Emsley, P. R. Evans, R. M. Keegan, E. B. Krissinel, A. G. Leslie, A. McCoy, S. J. McNicholas, G. N. Murshudov, N. S. Pannu, E. A. Potterton, H. R. Powell, R. J. Read, A. Vagin, K. S. Wilson, *Acta. Cryst.* **2011**, *D67*, 235.

[11] P. Emsley, B. Lohkamp, W. G. Scott, K. Cowtan, *Acta Cryst.* **2010**, *D66*, 486.

[12] A. Vagin, R. A. Steiner, A. A. Lebedev, L. Potterton, S. McNicholas, F. Long, G. N. Murshudov, *Acta Cryst*. **2004**, *D60*, 2284.

[13] V. B. Chen, W. B. Arendall, J. J. Headd, D. A. Keedy, R. M. Immormino, G. J. Kapral, L. W. Murray, J. S. Richardson, D. C. Richardson, *Acta Cryst*. **2010**, *D66* (Pt 1), 12.

[14] R. Green, A. Presta, Z. Gasyna, M. J. Stillman, *Inorg. Chem.* **1994**, *33*, 4159.

[15] D. E. K. Sutherland, M. J. Stillman, *Metallomics* **2011**, *3*, 444.

[16] X. Chen, H. Hua, K. Balamurugan, X. Kong, L. Zhang, G. N. George, O. Georgiev, W. Schaffner, D. P. Giedroc, *Nucleic Acid Res*. **2008**, *36*, 3128.

[17] F. Xie, D. E. K. Sutherland, M. J. Stillman, M. Y. Ogawa, *J. Inorg. Biochem.* **2010**, *104*, 261.
